# Effects of pharmacological and nonpharmacological treatments on brain functional magnetic resonance imaging in Alzheimer’s disease and mild cognitive impairment: a critical review

**DOI:** 10.1186/s13195-018-0347-1

**Published:** 2018-02-20

**Authors:** Elisa Canu, Elisabetta Sarasso, Massimo Filippi, Federica Agosta

**Affiliations:** 1 Neuroimaging Research Unit, Institute of Experimental Neurology, Division of Neuroscience, San Raffaele Scientific Institute, Vita-Salute San Raffaele University, Via Olgettina, 60, 20132 Milan, Italy; 2 Department of Neurology, Institute of Experimental Neurology, Division of Neuroscience, San Raffaele Scientific Institute, Vita-Salute San Raffaele University, Milan, Italy; 3 Laboratory of Movement Analysis, Division of Neuroscience, San Raffaele Scientific Institute, Vita-Salute San Raffaele University, Milan, Italy

**Keywords:** Alzheimer’s disease (AD), Mild cognitive impairment (MCI), Pharmacological treatments, Nonpharmacological treatments, Functional magnetic resonance imaging (MRI), Training, Cognition

## Abstract

**Background:**

A growing number of pharmacological and nonpharmacological trials have been performed to test the efficacy of approved or experimental treatments in Alzheimer disease (AD) and mild cognitive impairment (MCI). In this context, functional magnetic resonance imaging (fMRI) may be a good candidate to detect brain changes after a short period of treatment.

**Main body:**

This critical review aimed to identify and discuss the available studies that have tested the efficacy of pharmacological and nonpharmacological treatments in AD and MCI cases using task-based or resting-state fMRI measures as primary outcomes. A PubMed-based literature search was performed with the use of the three macro-areas: ‘disease’, ‘type of MRI’, and ‘type of treatment’. Each contribution was individually reviewed according to the Cochrane Collaboration’s tool for assessing risk of bias. Study limitations were systematically detected and critically discussed. We selected 34 pharmacological and 13 nonpharmacological articles. According to the Cochrane Collaboration’s tool for assessing risk of bias, 40% of these studies were randomized but only a few described clearly the randomization procedure, 36% declared the blindness of participants and personnel, and only 21% reported the blindness of outcome assessment. In addition, 28% of the studies presented more than 20% drop-outs at short- and/or long-term assessments. Additional common shortcomings of the reviewed works were related to study design, patient selection, sample size, choice of outcome measures, management of drop-out cases, and fMRI methods.

**Conclusion:**

There is an urgent need to obtain efficient treatments for AD and MCI. fMRI is powerful enough to detect even subtle changes over a short period of treatment; however, the soundness of methods should be improved to enable meaningful data interpretation.

## Background

Alzheimer’s disease (AD) is a devastating neurodegenerative disease and the most prevalent form of dementia [1]. There is an urgent need to identify effective treatments that may improve cognitive function in subjects with manifest or prodromal AD, and in people at risk of developing the disease, such as those with mild cognitive impairment (MCI). Currently there are two classes of drugs approved for the treatment of AD: the cholinesterase inhibitors, which are licensed for the treatment of mild-to-moderate AD, and memantine for moderate-to-severe disease stages [2]. These treatments have been demonstrated to be able to slow down the course of the disease but they cannot modify progression nor prevent onset [2]. Although no new therapeutics have been approved for AD in over 10 years, a substantial number of compounds thought to reduce amyloid and/or tau deposition are currently being testing [2]. The growing social emergency represented by AD and the lack of medical treatments able to modify the disease course have kindled interest in nonpharmacological therapies, such as cognitive stimulation, aerobic physical exercise, music therapy, and diet, with the aim of optimizing cognitive and functional skills and improving patient quality of life [3].

Numerous clinical trials have been performed to explore the efficacy of pharmacological and nonpharmacological treatments on cognitive and/or behavioral symptoms in AD and MCI patients. In clinical trials, outcome measures are typically performance-based instruments or structured surveys of clinician/caregiver impression of change [4]. Although the efficacy of treatments for AD and MCI must ultimately be demonstrated using clinically meaningful outcome measures, such trials will likely require hundreds of patients studied for medium term periods [5]. Thus, surrogate markers of efficacy with less variability than clinical assessments are needed to reduce the number of subjects. These markers may also be particularly valuable in the early phase of drug development to detect a preliminary “signal of efficacy” over a shorter time period.

Given the growing body of evidence that alterations in synaptic function are present very early in the course of the neurodegenerative disease process [6, 7], functional magnetic resonance imaging (fMRI) has been shown to be particularly useful for detecting early alterations in brain function and may be a critical marker for the detection of physiological changes over a short interval [8]. Specifically, fMRI may be valuable in evaluating acute and subacute effects of therapeutic interventions by showing how they modulate targeted circuits [9]. Using fMRI, the efficacy of treatments on brain function can be revealed by task-based or task-free (resting-state) approaches. By modeling cognitive paradigms, task-based fMRI explores cerebral functioning while the subject is performing specific activities that can mimic the actual difficulties occurring in daily life. A number of pioneering task-based fMRI studies have identified reduced activation in hippocampal and parahippocampal regions during episodic memory tasks in patients with AD [10–13] and, less consistently, both medial temporal lobe decreased and increased activation in patients with MCI [11, 12, 14–18]. In addition, resting-state fMRI has the potential to detect subtle functional abnormalities in brain networks supporting complex cognitive processes that are progressively impaired over the course of AD. At present, several studies of AD patients have demonstrated alterations of the default mode network (DMN) and other resting-state networks related to cognitive functions [19–21]. Compared to task-based approaches, resting-state imaging has the advantage of avoiding performance-related variability and is also less complicated to acquire and standardize [22].

The aim of this manuscript is to review studies that have tested pharmacological or nonpharmacological treatments in AD and MCI patients by using task-based or resting-state fMRI measures as primary outcomes. Furthermore, from a critical point of view, we explore the factors that could act as bias while verifying the efficacy of a treatment. Finally, we offer practical suggestions that could be useful in future studies.

## Methods

### Formal literature review research

A formal literature review was conducted on Medline in two separate sections, one for pharmacological and the other for nonpharmacological studies. In all cases, the research was performed on relevant articles (and their references) published in peer-reviewed journals before 20 March 2017 and with the use of three macro-areas, such as ‘disease’, ‘type of MRI’, and ‘type of treatment’. The disease has been searched with the single term ‘mild cognitive impairment’ or ‘MCI’ in the title and abstract only; or with the Mesh term ‘Alzheimer’s disease’ or with the same single term in the title and abstract only. The type of MRI was searched with the single terms ‘functional MRI’ or ‘fMRI’ or ‘functional connectivity’.

#### Pharmacological studies

The type of treatment was searched with the Mesh term ‘Therapeutics’ or the single terms ‘treatment’ or ‘pharmacological treatment’. The final search line was the following: ((((((Alzheimer Disease[MeSH Term]) OR alzheimer's disease[Title/Abstract]) OR MCI[Title/Abstract]) OR mild cognitive impairment[Title/Abstract])) AND (((functional mri) OR fmri) OR functional connectivity)) AND (((Therapeutics[MeSH Term]) OR treatment) OR pharmacological treatment).

#### Nonpharmacological studies

The type of treatment was searched with the Mesh term ‘Physical Therapy Modalities’ or ‘Exercise Therapy’ or the single terms ‘physical therapy’ or ‘motor rehabilitation’ or ‘physical training’ or ‘physical therapy’ or ‘exercise training’ or ‘physical exercise’ or ‘cognitive exercise’ or ‘cognitive rehabilitation’. The final search line was the following: (((((("Alzheimer Disease"[Mesh]) OR alzheimer's disease[Title/Abstract]) OR MCI[Title/Abstract]) OR mild cognitive impairment[Title/Abstract])) AND (((functional mri) OR fmri) OR functional connectivity)) AND ((((((((((("Exercise Therapy"[Mesh]) OR "Physical Therapy Modalities"[Mesh]) OR physical exercise) OR exercise training) OR physical therapy) OR physical training) OR motor rehabilitation) OR cognitive exercise) OR cognitive rehabilitation) OR cognitive training) OR cognitive stimulation).

### Critical review

Each original contribution was individually reviewed according to the Cochrane Collaboration’s tool for assessing risk of bias [23]. This tool provides criteria for judging the risk of bias in experimental designs testing the efficacy of treatments [23]. Each selected article was independently judged by two reviewers (EC and ES) according to seven categories: 1) random sequence generation; 2) allocation concealment; 3) blinding of participants and personnel; 4) blinding of outcome assessment; 5) short-term incomplete outcome data; 6) long-term incomplete outcome data; 7) and selective reporting [23]. The assessment was achieved by assigning a judgment of ‘low risk’ of bias when bias was absent or considered unlikely to have altered the results, ‘high risk’ of bias when the potential for bias weakened confidence in the results, and ‘unclear risk’ when there was some doubt about the effect of bias on the results due to insufficient information. When no agreement was reached between the two reviewers, the specific article was further discussed with a third reviewer (FA) for a final judgment. Further technical biases were identified by the reviewers according to their expertise in neuroimaging, neurology, neuropsychology, and physiotherapy fields and were discussed in appropriate sessions.

## Results

### Pharmacological studies

**Fig. 1:**
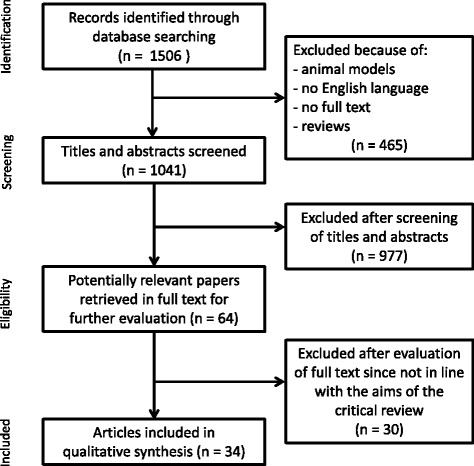
Flowchart of literature review process (pharmacological functional magnetic resonance imaging studies)

We obtained 1506 articles. Through title and/or abstract reading, we excluded review articles, articles that did not directly look at the treatment effect on fMRI measures, animal model studies, and articles written in non-English languages. We included 34 pharmacological studies (Fig. 1 and Table 1). Twelve studies were on MCI patients, 21 on AD patients (16 on mild AD, 4 on mild-to-moderate AD, 1 on moderate AD), and one included both mild AD and MCI cases. Twelve studies were randomized controlled trials while the others had a nonrandomized or an observational design.

**Table 1:** Detailed findings of pharmacological fMRI studies

Reference	Treatment	Design	Sample	fMRI protocol/scan timing	Outcome measures	Clinical findings	Direction fMRI changes	Brain areas involved	Clinical-fMRI relationship
Bakker et al., 2015 [40]	Levetiracetam (different doses: 62.5 mg twice/day, 125 mg twice/day, and 250 mg twice/day) and placebo	RCT double-blind for patients and single-blind for HC	17 HC 54 MCI	Three-choice recognition memory task Pre-/post-treatment	Task-related medial temporal, temporal-polar, and hippocampal functional activity changes; performance improvement at fMRI task and cognitive assessment	Improvement on recognition memory task in the group on low-dose treatment. No changes at the BSRT, Verbal Pair Associate test, or BVRT	Decreased	Post-treatment vs placebo: L CA3 and DG of hippocampus	Decreased activity; higher memory performance during task
							Increased	Post-treatment vs placebo:L entorhinal cortex	
Bakker et al., 2012 [41]	Levetiracetam (125 mg twice/day) and placebo	RCT double-blind for patients and single-blind for HC	17 HC 17 MCI	Three-choice recognition memory task Pre-/post-treatment	Task-related hippocampal functional activity changes; performance improvement at fMRI task and cognitive assessment	Improvement on recognition memory task. No changes at the BSRT, Verbal Pair Associate test, and BVRT	Decreased	Post-treatment vs placebo: L CA3 and DG of hippocampus	Decreased activity; higher memory performance during task
Bentley et al., 2008 [26]	Physostigmine (infusion at a rate of 1 mg/1 h) and placebo (an equivalent volume of saline), in both groups 25 min prior to scan	NRCT double-blind	17 HC 16 mild AD	Visuo-attentional task Post-treatment	Task-related parietal functional activity changes; performance improvement at fMRI task	Improvement on RT for the ‘deeper’ task in AD	Increased	Group X time, treated vs placebo: R precuneus and posterior parahippocampal cortex; R parietal and PFC	–
							Decreased	Group X time, treated vs placebo: R fusiform gyrus	
Bentley et al., 2009 [27]	Physostigmine (infusion at a rate of 1 mg/1 h) and placebo (an equivalent volume of saline), in both groups 25 min prior to scan	NRCT double-blind	18 HC 13 mild AD	Face-encoding task Post-treatment	Task-related fusiform functional activity changes and their relationship with performance improvement at fMRI task	Task-independent (‘shallow’ vs ‘deeper’) improvement in confident memory	Increased	Group X time, treated vs placebo: Bilateral fusiform cortex	Increased activity; higher face recognition post-scanning
Blautzik et al., 2016 [55]	Galantamine (6-month treatment: 8 mg/day for the first month; 16 mg/day for the second month; 24 mg/day for the other months) or placebo, followed by 6 months galantamine (24 mg/day) – open label period	RCT double-blind and open-label	11 HC 13 mild-moderate AD	RS fMRI At baseline At 6 months At 12 months	DMN functional connectivity changes; performance improvement at cognitive assessment	No changes at the CEREAD	Increased	Post-treatment vs HC (12-month follow-up):Posterior DMN (PCC, precuneus, L > R); Post-treatment vs placebo (12-month follow-up):Hippocampal sub-component (anterior division of hippocampus, R > L)	–
Bokde et al., 2016 [47]	Rivastigmine (3-month treatment: 3 mg/day for the first month; 6 mg/day for the second month; 9 mg/day for the third month) or placebo, followed by 9 months rivastigmine (9 mg/day) – open label period	RCT double-blind and open-label	12 MCI	Face- and location-matching task At baseline At 3 months At 6 months	Task-related whole-brain functional activity changes and performance improvement at cognitive assessment	After 3 and 6 months: lower performances at the verbal fluency; stable performances at the CERAD and at the task	Increased	Pre-/post-treatment (3-month follow-up): Face-matching task: bilateral lingual and fusiform gyrus, L angular gyrus and cerebellum. Location matching task: L inferior temporal gyrus, R precuneus, R angular and inferior frontal gyri	–
								Pre-/post-treatment (6-month follow-up): Location matching task: R inferior parietal and supramarginal gyrus, L precuneus and paracentral lobule, L medial frontal gyrus	
Bokde et al., 2009 [32]	Galantamine (3-month treatment: 8 mg/day for the first month; 16 mg/day for the second month; 24 mg/day for the last month)	Case series	5 mild AD	Face- and location-matching task Pre-/post-treatment	Task-related ventral and dorsal visual pathway changes; performance improvement at fMRI task and cognitive assessment	No changes at the task or at the CEREAD	Decreased	Pre-/post-treatment:Location-matching task: bilateral dorsal pathway (from occipital to parietal and frontal cortices)	–
Dhanjal et al., 2013 [29]	Donepezil (6-week treatment: 5 mg/day for the first 2 weeks; 10 mg until the end of the study)	Case series	9 mild AD	Auditory sentence encoding and retrieval with auditory working memory suppressors Pre-/post-treatment	Task-related primary auditory, ventro-lateral temporal, pars triangularis and angular gyri functional activity changes; performance improvement at fMRI task	Increased percentage of retrieved trials during task	Increased	Pre-/post-treatment: L anterior ventral temporal cortex and pars triangularis	–
Dhanjal et al., 2014 [30]	Donepezil (6-week treatment: 5 mg/day for the first 2 weeks; 10 mg until the end of the study)	Cohort study	18 HC 18 mild AD	Auditory sentence encoding and retrieval with auditory working memory suppressors Pre-/post-treatment	Task-related functional activity changes within the executive and salience networks; performance improvement at fMRI task	Increased percentage of retrieved trials during task	Increased	Pre-/post-treatment: Fronto-parietal executive network: L lateral posterior parietal cortex and lateral frontal cortex. Higher-order cortex: L parahippocampal gyrus and anterior ventral temporal cortex	–
Goekoop et al., 2004 [39]	Galantamine (oral intake, single dose: 8 mg; and after prolonged exposure: 4 mg day 1, 8 mg next 4 days, 4 mg on day 6). Washout period: 2 days	Cross-over	28 MCI	Episodic face-encoding and N-letter back task Pre-/post-treatment	Task-related whole-brain functional activity changes	N-letter back: task accuracy increased and latency decreased, mainly after single dose intake	Increased	Pre-/post-treatment (prolonged exposure): Face encoding: L middle frontal and occipital cortices, L posterior hippocampus and R anterior cingulate cortex. N-letter back: R precuneus and middle frontal cortex	–
Goekoop et al., 2006 [31]	Galantamine (acute (8 mg) and prolonged 5 days exposure (4 mg the first day, 8 mg the following 4 days, 4 mg the last day))	Cross-over	18 mild AD 28 MCI	Face-recognition task Pre-/post-treatment	Task-related whole-brain functional activity changes	No changes at the task	Increased	Pre-/post-treatment (acute exposure): MCI: L PCC, anterior and temporal lobe, L superior parietal, R frontal lobe and cerebellum. AD: vermis of cerebellum, R inferior temporal and parahippocampal gyri	–
							Decreased	Pre-/post-treatment (prolonged exposure): MCI: bilateral superior frontal cortices, L PCC, R middle frontal gyrus. AD: R parahippocampal cortex	
Goveas et al., 2011 [50]	Donepezil (3-month treatment: 5 mg/day for 4 weeks; 10 mg/day until the end of the study)	Cohort study	14 HC 18 mild AD	RS fMRI, seed-based (hippocampus) connectivityPre-/post-treatment	Hippocampal functional connectivity changes; performance improvement at cognitive assessment	Improvement on ADAS-cog but not on MMSE	Increased	Pre-/post-treatment: Positively correlated hippocampal functional connectivity network: L middle frontal and precentral gyri, L parahippocampus, insula and thalamus, R PCC	Increased hippocampal connectivity strength in the L dorsolateral PFC and middle frontal gyrus; improvement on ADAS-cog
							Decreased	Pre-/post-treatment: Negatively correlated hippocampal functional connectivity network: L inferior parietal cortex/supramarginal gyrus, L posterior middle temporal gyrus, and R dorsolateral PFC	
Griffanti et al., 2016 [52]	Donepezil (12-week treatment: 5 mg/day for the first 4 weeks, followed by 10 mg/day until the end of the study)	Case series	18 mild-moderate AD	RS fMRIPre-/post-treatment	Relationship between whole-brain functional connectivity changes and performance improvement at cognitive assessment	Greater improvement on MMSE and MoCA in responders compared to nonresponders	Increased	Pre-/post-treatment: Orbitofrontal network: precuneus, PCC and R dorsolateral frontal cortex (responders > nonresponders)	Increased connectivity of anterior and posterior cingulate cortices, precuneus, and R dorsolateral frontal regions within the orbitofrontal network; improvement on MoCA
Grön et al., 2006 [38]	Galantamine (4 mg twice a day for 7 days)	Case series	10 MCI	Spatial navigation taskPre-/post-treatment	Task-related hippocampal functional activity changes and performance improvement at cognitive assessment	Improvement on verbal episodic memory but not at the task	Increased	Pre-/post-treatment: R middle occipital and temporal gyri, R PCC, R hippocampus and parahippocampal gyrus; L anterior hippocampus	–
Haller et al., 2014 [45]	Caffeine (one capsule containing caffeine 200 mg or placebo) 30 min before testing	NRCT double-blind	15 HC 13 MCI	2-back (vs 0-back) working memory taskPre-/post-treatment	Task-related whole-brain functional activity changes	No effect on task RT neither on accuracy	Increased	Post-treatment vs placebo: Task-related: bilateral striatum, temporal and parietal cortices. TICA: L working memory network including PFC, supplementary motor area, ventral premotor and parietal cortices	–
Kircher et al., 2005 [28]	Donepezil (10-week treatment: 5 mg/day for the first 4 weeks; 10 mg/day until the end of the study)	Cohort study	10 HC 10 mild-moderate AD	Face memory encoding taskPre-/post-treatment	Task-related fusiform gyrus functional activity	Improvement on ADAS-cog total score and on the memory subscale. No changes at the task	Increased	Pre-/post-treatment/Post-treatment vs HC: R fusiform gyrus	–
Li et al., 2012 [51]	Donepezil (12-week treatment: 5 mg/day for the first 4 weeks; 10 mg/day until the end of the study)	Case series	12 mild AD	RS fMRI, seed based (MCC and PCC) connectivityPre-/post-treatment	MCC and PCC functional connectivity changes; cerebral blood flow changes; performance improvement at cognitive assessment	Improvement on ADAS-cog but not on MMSE, NPI, or IADL	Increased	Pre-/post-treatment: Middle cingulate and PCC network connectivity	Increased connectivity between the middle cingulate cortex and the ventral anterior cingulate cortex and PFC; and between the PCC and the ventral anterior cingulate cortex-changes in ADAS-cog
Lorenzi et al., 2011 [56]	Memantine (6-month treatment: 5 mg/day, increasing by 5 mg/day to a final dose of 20 mg/day) or placebo	RCT double-blind	15 moderate AD	RS fMRI Pre-/post-treatment	DMN functional connectivity changes; performance improvement at cognitive assessment	No changes at the cognitive assessment	Increased	Pre-/post-treatment/Group X time, treated vs placebo: R precuneus and calcarine gyrus within DMN	–
McGeown et al., 2010 [36]	Donepezil (20-week treatment: 10 mg/day)	Cohort study	9 HC 12 mild AD	Semantic association and N-back (1-back) task Pre-/post-treatment	Task-related whole-brain functional activity changes; performance improvement at fMRI task	No changes at the cognitive assessment (including ADAS-cog, NPI and ADL) or at the task	Decreased	Pre-/post-treatment: Semantic association: L superior parietal, middle temporal, medial and inferior frontal gyrus, and R superior temporal gyrus. Working memory: L caudate, L middle and superior temporal gyri, and R inferior frontal gyrus. Post-treatment vs HC: Semantic association: bilateral middle frontal gyrus, R superior occipital, cuneus and anterior cingulate cortex. Working memory: L thalamus, L parahippocampal gyrus, R inferior frontal gyrus	Increased activity in non-task relevant regions (such as bilateral inferior parietal lobe, PCC and precuneus); higher accuracy at the semantic association task
McGeown et al., 2008 [34]	Rivastigmine (20-week treatment: 6 mg twice/day)	Cohort study	9 HC 11 mild AD	Semantic association and N-back (1-back) task Pre-/post-treatment	Task-related whole-brain functional activity changes; performance improvement at cognitive assessment	Improvement on ADAS-cog. No further changes at the cognitive assessment or at the task	Increased	Pre-/post-treatment: Semantic association: bilateral middle frontal and paracentral gyri, parahippocampal and fusiform gyri. Working memory: R superior, middle, medial and inferior frontal gyrus, and R precentral gyrus. Post-treatment vs HC: Semantic association: R inferior frontal and L anterior cingulate cortex. Working memory: R middle frontal, postcentral and supramarginal gyri	–
							Decreased	Pre-/post-treatment: Working memory: L middle frontal, precentral and cingulate gyrus, L insula and thalamus. Post-treatment vs HC: Working memory: L PCC and angular gyrus	
Miettinen et al., 2011 [24]	A single oral dose of rivastigmine (3 mg, acute); and 1.5 mg of rivastigmine twice a day for 4 weeks (chronic); a single oral dose of placebo	NRCT double-blind	20 mild AD	Face recognition memory task Post-treatment	Task-related whole-brain functional activity changes and their relationship with baseline cognitive assessment	No changes at the task	Increased	Post-treatment vs placebo (acute): bilateral PFC, R middle and superior temporal gyrus. Post-treatment vs placebo (chronic): bilateral PFC, L middle temporal and anterior cingulate cortices, and L parietal gyrus	Increased PFC activity after chronic treatment; poorer MMSE at baseline
Pa et al., 2013 [42]	Donepezil (3-month treatment: 5 mg/day for 1 month and 10 mg/day for 2 months) or placebo	RCT double-blind	27 MCI	Face recognition task Pre-/post-treatment	Task-related prefrontal, parietal and hippocampal functional activity changes; performance improvement at cognitive assessment	Improvement on task RT and accuracy. No changes at the cognitive assessment	Increased	Group X time, treated vs placebo: L fusiform face area and its connectivity with R hippocampus and inferior frontal junction	Increased connectivity between L fusiform face and R hippocampus; reduced RT for face recognition in treated patients
Petrella et al., 2009 [44]	Donepezil (12- or 24- week treatment: 5 mg/day for 42 days, followed by 10 mg/day until the end of the study)	RCT double-blind	13 MCI	Novel face visual memory task Pre-/post-treatment	Task-related whole-brain functional activity changes; performance improvement at cognitive assessment	No improvement at the cognitive assessment or at the task	Increased	Post-treatment vs placebo: Bilateral dorsal e ventrolateral PFC. Group X time,treated vs placebo: L inferior frontal gyrus	–
Risacher et al., 2013 [43]	Donepezil (3-month treatment: 5 mg/day for 4 weeks, 10 mg/day until the end of study)	NRCT open-label	20 HC 18 MCI	Verbal episodic encoding task Pre-/post-treatment	Task-related whole-brain functional activity changes and their relationship with patient performances at cognitive assessment before and after treatment	Improvement on CVLT. Mild accuracy decline during task	Increased	Group X time/treated vs HC: R hippocampus and parahippocampal gyrus, R middle frontal gyrus. Increased deactivation of the medial parietal lobe	Changes on medial parietal lobe activity-changes in CVLT. Increased connectivity of the L frontal lobe and L caudate; improved task accuracy
Rombouts et al., 2002 [25]	Single dose (3 mg) of rivastigmine, 3 h before the first *vs* the second scanning	NRCT single-blind	11 mild AD	Face encoding and working memory task Pre-/post-treatment	Task-related whole-brain functional activity changes	No changes at the task	Increased	Post-treatment vs placebo: Face encoding: bilateral fusiform gyrus. Simple working memory: L middle and superior frontal gyrus. Increased working memory load: L middle frontal gyrus, R inferior and superior frontal gyrus.	–
							Decreased	Post-treatment vs placebo: Increased working memory load: R middle and superior frontal gyrus	
Saykin et al., 2004 [46]	Donepezil (5 mg/day for 4 weeks; 10 mg/day for 5.67 ± 1.66 weeks on average)	NRCT open-label	9 HC 9 MCI	Auditory N-back task Pre-/post-treatment	Task-related whole-brain functional activity changes; performance improvement at cognitive assessment and fMRI task	Improvement on accuracy during task and on TMT-B. Reduction of subjective cognitive concerns	Increased	Group X time, treated vs HC: L dorsolateral PFC and L superior frontal cortex	Increased activity of the L anterior prefrontal-improved task accuracy
Shanks et al., 2007 [35]	Galantamine (20-week treatment: 16 mg twice/day)	Cohort study	9 HC 9 mild AD	Semantic association and target detection task Pre-/post treatment	Task-related frontal and parieto-temporal functional activity changes	No improvement at the cognitive assessment or at the tasks. Increased awareness in patient self-assessment with respect to problems during daily activities	Increased	Pre-/post-treatment: Semantic association: L paracentral lobule, L caudate and R lingual gyrus. Target detection: bilateral postcentral, L inferior parietal lobule. Post-treatment vs HC: Semantic association: bilateral superior temporal gyri and insula, R medial frontal gyrus, L inferior frontal. Target detection: bilateral middle frontal, L superior temporal and precuneus	–
Solé-Padullés et al., 2013 [49]	Donepezil (3-month treatment: 5 mg/day for 1 month and 10 mg/day for 2 months) or no treatment	RCT single-blind	15 mild-moderate AD	RS fMRI andvisual scene encoding task Pre-/post-treatment	RS whole-brain functional connectivity and task-related activity changes; performance improvement at fMRI task	Improvement on semantic fluency. No further changes at the cognitive assessment or at the task	Increased	Post-treatment vs untreated: R parahippocampal gyrus within the DMN. No task-related changes were observed	–
Thiyagesh et al., 2010 [33]	Donepezil (23-week treatment: 5 mg/day)	Cohort study	11 HC 10 mild AD	Visuospatial tasks Pre-/Post-treatment	Task-related functional activity changes in brain regions subtending visuospatial abilities	Improvement on MMSE, ADAS-cog, and Present Functioning Questionnaire. No changes at the task	Increased	Pre-/post-treatment: L precuneus	Increased activity of the L precuneus-improvement atthe Present Functioning Questionnaire
Venneri et al., 2009 [37]	AchEI treatment (20-week treatment: at the maximum guideline-recommended dosage)	Cohort study	9 HC 26 mild AD	Semantic association and N-back (1-back) task Pre-/post-treatment	Task-related whole-brain functional activity changes and performance improvement at cognitive assessment in responders compared with nonresponders	Improvement of the responders on ADAS-cog. No further changes at the cognitive assessment or at the task	Increased	Pre-/post-treatment/Group X time, responders vs nonresponders: Semantic association: bilateral inferior and medial frontal gyri, L precentral and postcentral gyri, L insula, middle temporal and inferior parietal gyri and anterior cingulate cortex; R inferior temporal gyrus, precuneus and caudate. Working memory: R precentral, precuneus, inferior parietal and thalamus, L inferior and superior frontal gyrus	Increased activity of the L frontal cortex during the semantic association task; poorer performance at the baseline semantic fluency
Wang et al., 2014 [54]	Stable dose of AchEIs (donepezil, rivastigmine, or galantamine) for at least 15 days and for almost 18 months	Case-control	25 mild treated AD 19 mild untreated AD	RS fMRI Post-treatment	Functional connectivity changes and interaction with the APOE genotype	–	Increased	Pre-/post-treatment/ApoEε4 treated vs ApoEε4 untreated: Greater composite scores in dorsal attention, control and salience networks	–
Zaidel et al., 2012 [53]	Donepezil (8-week treatment: 5 mg/day for 28 days; 10 mg/day until the end of the study)	Case series	11 mild AD	RS fMRI, L hemisphere seed-based connectivity Pre-/post-treatment	RS functional changes in the interhemispheric connectivity	–	Increased	Pre-/post-treatment: L-R dorsolateral PFC	–
Zhang et al., 2016 [57]	Bushen capsule (24-month treatment: 4 capsules 3 times a day) or placebo	RCT double-blind	60 MCI	RS fMRI At baseline At 12 months At 24 months	DMN functional connectivity and performance improvement at cognitive assessment, and their relationship	Improvement on MMSE, RAVLT, and digit span	Increased	Group X time, treated vs placebo:R precuneus within the DMN	No relationship was observed between connectivity and cognitive changes
Zhang et al., 2014 [48]	CCRC (3-month treatment: 3 capsules per day) or placebo	RCT double-blind	39 MCI	N-back (0-1-and 2 back) working-memory task Pre-/Post-treatment	Task-related whole-brain functional activity changes; performance improvement at cognitive assessment	Improvement on MMSE and digit span. No further changes on other cognitive scores or on task	Increased	Group X time, treated vs placebo and vs HC: Increased negative activation of L PCC and R fusiform gyrus	Increased negative activity in L PCC; changes on MMSE and digit span scores

*AchEI* acetyl-cholinesterase inhibitor, *AD* Alzheimer’s disease, *ADAS-cog* Alzheimer's Disease Assessment Scale-cognitive subscale, *ADL* activities of daily living, *APOE* apolipoprotein E, *BSRT* Buschke Selective Reminding Test, *BVRT* Benton Visual Retention Test, *CCRC* Compound Congrongyizhi Capsule, *CEREAD* Consortium to Establish a Registry for Alzheimer's Disease, *CVLT* California Verbal Learning Test, *DG* dentate gyrus, *DMN* default mode network, *fMRI* functional MRI, *HC* healthy controls, *IADL* instrumental activities of daily living, *L* left, *MCC* middle cingulate cortex, *MCI* mild cognitive impairment, *MMSE* Mini mental state examination, *MoCA* The Montreal Cognitive Assessment, *NPI* Neuropsychiatric Inventory, *NRCT* nonrandomized controlled trial, *PCC* posterior cingulate cortex, *PFC* prefrontal cortex, *R* right, *RAVLT* Rey auditory verbal learning test, *RCT* randomized controlled trial, *RS fMRI* resting state functional MRI, *RT* reaction time, *shallow* low-demanding, *TICA* tensorial-independent component analysis, *TMT-B* Trail Making Test, part B

#### Summary

As expected, the effect of acetyl-cholinesterase inhibitors (AchEI) has been investigated in the majority of studies (82%), followed by levetiracetam (6%), memantine (3%), caffeine (3%), and Chinese medicines such as the Compound congrongyizhi and the Bushen capsules (6%). In general, treatments lasted from a day (acute) to 6 months. Only in one study did the authors observe the effect of the proposed treatment over 24 months. The adopted fMRI approach was: task-based fMRI in 74% of studies, using memory (44%, such as encoding, retrieval, recognition and/or matching tasks), visual attention (3%), visuospatial or spatial navigation (6%), N-back (18%), or semantic association paradigms (3%); resting-state fMRI in 23% of studies; and both resting-state fMRI and visual encoding paradigms in the remaining 3%. fMRI studies showed positive effects of cognitive enhancing drugs on brain activation during cognitive task performance or the resting state in patients with AD and MCI. Both acute and prolonged exposure to pharmacological therapies were associated with fMRI changes in AD-specific and non-AD regions. In the majority of the studies, these changes were in parallel with improved fMRI task performance and global cognition assessed with a formal neuropsychological assessment outside the scanner. However, due to the heterogeneity of pharmacological treatment, dosage, and cognitive paradigms used for fMRI tasks, a generalization of the results is challenging.

In mild AD, a single dose (3 mg) of rivastigmine [24, 25] or infusion of physostigmine [26, 27] compared to placebo were associated with a greater activation of the right precuneus and parahippocampal gyrus [26], bilateral fusiform cortex [25, 27], and prefrontal areas [24] during face-recognition memory paradigms, which correlated with improved task performance [24, 27]. Using a similar paradigm in mild-moderate AD, increased right fusiform gyrus was observed after 10 weeks of donepezil [28]. During a task assessing the auditory process of verbal memory in mild AD, the activity was increased in mild AD patients in the left temporal cortex, parahippocampal gyrus, and frontoparietal executive network, together with an increase of successfully retrieved trials after 6 weeks of donepezil [29, 30]. During a face-recognition task, both increased activation after acute (8 mg) and decreased activation after prolonged (5 days) galantamine exposure were observed in parahippocampal regions in mild AD [31]. In mild AD patients, 3 months of treatment with galantamine reduced the fMRI signal within the dorsal pathway during a location-matching test [32]. Most studies which investigated the effect of prolonged treatment exposure showed that mild AD patients “normalized” the fMRI activity to the level of controls at baseline in AD-crucial regions after about 20 weeks of donepezil [33], rivastigmine [34], and galantamine [35] treatments, in parallel with improved global cognition and task performance [33, 34]. However, not all studies found a correlation between fMRI changes and clinical improvement, e.g., McGeown et al. demonstrated a widespread pattern of decreased fMRI activity during semantic association and working memory tasks after 20 weeks of donepezil but higher accuracy in task performance was associated with increased recruitment in nontask-relevant regions [36]. Finally, fMRI changes were observed to be greater in AchEI “responders” [37].

In MCI patients, increased fMRI activity in hippocampus and parahippocampal regions were observed during a spatial navigation task after only 7 days of galantamine treatment [38] as well as during face encoding after 6 days exposure to the same therapy [39]. A stabilization of fMRI hippocampal activity (decreased to the level of healthy controls) during a memory recognition task was found after 2 weeks at low doses of levetiracetam, with parallel improvement in patient memory performance [40, 41]. During a face-recognition task, increased activation after acute (8 mg) and decreased activation after prolonged (5 days) galantamine exposure were observed in posterior cingulate cortex (PCC), superior parietal regions, and frontal cortex in MCI patients [31]. In MCI patients, better task performance, enhanced functional connectivity between the hippocampus and the fusiform face area during a face recognition fMRI task [42], and enhanced connectivity between the hippocampus and frontal and striatal regions during a verbal episodic encoding task [43] were observed after 3 months of treatment with donepezil. Increased inferior frontal fMRI activity was observed during face retrieval after 3 to 6 months of the same treatment [44]. Using working memory and location matching task paradigms in MCI patients, acute administration of caffeine [45], about 10 weeks of treatment with donepezil [46], 3 to 6 months of treatment with rivastigmine [47], and 3 months exposure to Compound Congrongyizhi Capsule [48] enhanced the functional activity in the frontoparietal pathway, with improved patient accuracy during the tasks [46].

Several resting-state fMRI studies reported increased functional connectivity after pharmacological treatments in mild-to-moderate AD patients. Increased connectivity was observed in the DMN [49], between the hippocampus and several cortical and subcortical regions [50], and between the PCC and prefrontal and parietal brain regions [51] after 3 months of donepezil, in parallel with an improvement in global cognitive scores [49–51]. In addition, increased resting-state connectivity was observed after 3 to 4 months of donepezil in non-DMN orbitofrontal [52] and dorsolateral prefrontal networks [53]. This effect was observed to be greater in apolipoprotein E ε4 carriers and to be present regardless of the kind of AchEI administered [54]. In mild-moderate AD, increased resting-state functional connectivity was also observed in the posterior and hippocampal DMN components after 12 months of galantamine [55] and in moderate-severe AD after 6 months of memantine [56]. Importantly, one study showed that 3 months of treatment with donepezil in mild AD cases was also associated with “restored”/stabilized hippocampal connectivity (i.e., decreased negative correlations) with cortical regions in the parietal, temporal, and frontal cortices [50]. In MCI patients, resting-state connectivity increased in the right precuneus within the DMN with parallel improvement in verbal and working memory after 24 months of treatment with Bushen capsules [57].

Although several studies showed both clinical and fMRI changes after pharmacological therapies (Table 1), none of them directly compared clinical and fMRI effect sizes in order to define the most powerful marker to monitor treatment efficacy.

#### Critical review

**Fig. 2:**
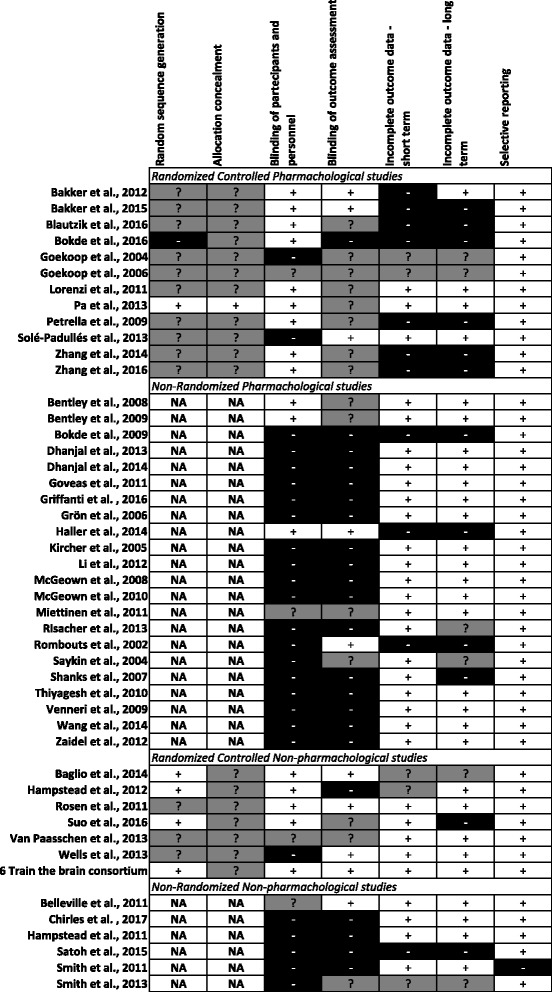
Judgments of articles according to the seven categories of the Cochrane Collaboration’s tool for assessing risk of bias. Positive marks denote low risk or no bias; negative marks denote high-risk bias; question marks denote unclear information. NA not applicable

According to the Cochrane Collaboration’s tool for assessing risk of bias, 12 studies (35%) were randomized but only one described clearly the randomization procedure and the allocation. Twelve studies (35%) declared the blindness of participants and personnel; for two studies (6%) this information was unclear, and the other 20 reports (59%) were unblinded. Five studies (15%) declared the blindness of outcome assessment; for 12 studies (35%) this information was unclear, and the other 17 (50%) were unblinded. Eleven studies (32%) presented more than 20% drop-outs at short- and/or long-term assessments leading to ‘high risk’ bias due to incomplete outcome data. All studies appropriately reported the primary and the secondary outcome measures of the investigation. A report of the final judgments for each selected article is shown in Fig. 2.

### Nonpharmacological studies

**Fig. 3:**
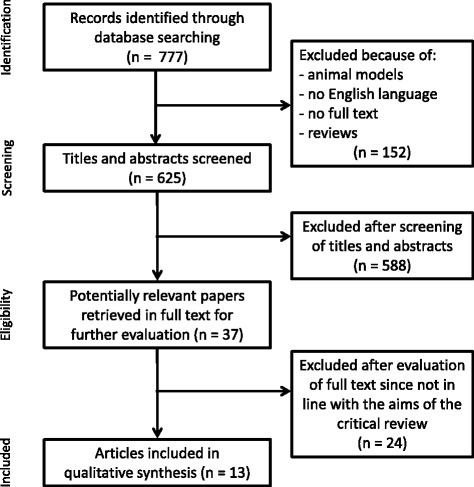
Flowchart of literature review process (nonpharmacological functional magnetic resonance imaging studies)

We obtained 777 articles and we excluded articles due to the same reasons reported above for the pharmacological studies. Two further articles were manually identified through the reference lists of the selected manuscripts. We included 13 nonpharmacological studies (Fig. 3 and Table 2), with 10 studies on MCI patients (five on cognitive-rehabilitation, three on physical rehabilitation, and two combined) and three on AD patients (two on cognitive-rehabilitation and one on combined cognitive-physical training—one on mild AD and two on mild-to-moderate AD). Seven studies were randomized controlled trials while the others had a nonrandomized or an observational design.

**Table 2:** Detailed findings of nonpharmacological fMRI studies

Reference	Treatment	Design	Sample	fMRI protocol/scan timing	Outcome measures	Clinical findings	Direction fMRI changes	Brain areas involved	Clinical-fMRI relationship
Baglio et al., 2015 [68]	MST focused on AD and caregivers. ADperformed 30 sessions involving reality orientation, cognitive exercises, physical, recreational and occupational activities (2.5 h/day, 3 days/week). Caregivers underwent an educational program to favor a long-term positive interaction with patients at home. Control group: AD receiving usual care	RCT single-blind	60 mild-moderate AD	Verbal fluency task Pre-/Post-training	Task-related whole-brain functional activity changes, performance improvement at cognitive assessment (ADAS-cog, FLSA, NPI, SF-36) and fMRI task, and their relationship	Improvement on NPI and on language and memory assessed with ADAS-Cog in MST relative to the control group after 10 weeks. No changes on functional status and physical well-being after 10 weeks. No further changes after 22 weeks of training. No improvement at the task	Increased	Group X time, training vs control:Bilateral superior temporal gyrus, R insula and thalamus	Increased brain activity; improvement at the ADAS-Cog total score
Belleville et al., 2011 [60]	Group episodic memory encoding and retrieval training (6 sessions/week of 120 min each) consisting of interactive imagery, face-name associations, hierarchical organization and semantic organization	NRCT single-blind	15 HC 15 MCI	Verbal memory encoding and retrieval task Pre-/Post-training	Task-related whole-brain functional activity changes; performance improvement at fMRI task; performance at the Côte-des-Neiges Computerized Memory Battery (immediate and delayed word recall)	Improvement on immediate and delayed word recall at the Côte-des-Neiges Computerized Memory Battery; performance improvement at the task for both encoding and retrieval	Increased	Pre-/post-training, MCI:Encoding: L superior temporal gyrus, insula and basal ganglia, R superior frontal and cerebellum. Retrieval: L postcentral, inferior parietal and superior temporal gyri, R superior temporal and middle frontal gyri, insula and precuneus	Increased activity of the R inferior parietal lobule; improved performance on delayed word recall
Chirles et al., 2017 [66]	Moderate intensity aerobic exercise on treadmill (12-week treatment, 30 min-walk, 4 times/week)	NRCT open-label	16 HC 16 MCI	RS fMRI, seed-based (PCC/precuneus) connectivity Pre-/Post-training	PCC/precuneus functional connectivity changes; performance improvement at cognitive and physical assessments	Improvement on mean intensity of training, rate of perceived exertion, VO2 peak and RAVLT	Increased	Pre-/post-training, MCI:Connectivity between PCC/precuneus and bilateral frontal and parietal, R temporal and insular cortices and L cerebellum. Group X time, MCI vs HC: connectivity between PCC/precuneus and inferior parietal lobule	–
Hampstead et al., 2011 [61]	Mnemonic strategy training using face-name associations (3 total sessions/2 weeks)	Case series	6 MCI	Face-name association task Pre-/Post-training	Task-related whole-brain functional activity changes; performance improvement at cognitive assessment	Improvement on memory performance during the task	Increased	Pre-/post-training, MCI: Bilateral medial frontal, medial parietal, medial occipital cortex, L frontal operculum, temporo-parietal cortex. The L middle temporal gyrus was the primary “driver” of activation (effective connectivity)	–
Hampstead et al., 2012 [62]	Mnemonic strategy training using object-location associations (3 total sessions/2 weeks) Control group: unspecific mnemonic training	RCT single-blind	16 HC 18 MCI	Object-location association task Pre-/Post-training	Task-related hippocampal functional activity changes; performance improvement at fMRI task; relationship between functional activity changes and performance improvement at fMRI task	No improvement at the task	Increased	Pre-/post-training, trained MCI: Encoding: L hippocampal body during both the trained and untrained stimuli. Retrieval: L hippocampal body and tail during the untrained stimuli. Group X time, trained MCI vs control: Retrieval: L hippocampal body and R hippocampus during trained stimuli; R hippocampal body during untrained stimuli	–
Rosen et al., 2011 [63]	Average of 2-month computer-based, cognitive training program focused on auditory verbal discrimination (100 min/day for 24 sessions). Control group, computer-based unspecific activities (90 min/day for 24 sessions)	RCT double-blind	12 MCI	Auditory-verbal task Pre-/Post-training	Task-related L hippocampal functional activity changes; performance improvement at fMRI task; performance at the RBANS	Improvement on memory assessed with the RBANS. No improvement at the task	Increased	Group X time, training vs control: L hippocampus	Increased activity L hippocampus-trend toward improvement at RBANS
Satoh et al., 2015 [59]	Singing training (6-month training, 1 session/week). Control group: AD who did not perform the training	NRCT open-label	20 mild-moderate AD	Karaoke and Pitch tasks Pre-/Post-training	Task-related whole-brain functional activity changes; performance improvement at the cognitive/behavioral assessment	Improvement on disability, behavior and reasoning assessed with DAD, NPI, and RCPM, respectively	Increased	Pre-/post-training, AD: R angular gyrus and L lingual gyrus	–
Smith et al., 2013 [65]	12-week moderate intensity treadmill training (44 total sessions: 30 min each session, 4 sessions/week)	NRCT open-label	18 HC 17 MCI (different subtypes)	Famous-name discrimination task Post-observation	Task-related whole-brain functional activity changes; performance improvement at the cognitive, physical assessments and at the fMRI task	Improvement on mean intensity of training, rate of perceived exertion, VO2 peak and RAVLT. No improvement at the task	Unchanged	No pre-/post-training or Group X time effect	–
Smith et al., 2011 [67]	Low-physical activity (≤ 2 days/week of low-intensity physical activity); High-physical activity (≥ 3 days/week of moderate to vigorous physical activity)	Case-control open-label	18 MCI	Famous-name discrimination task Pre-/Post-training	Task-related whole-brain functional activity changes; basal ganglia volume changes	–	Increased	Post-training/high vs low-physical activity: L caudate	–
Suo et al., 2016 [70]	26-week training (two sessions per week, each for 90 min). Four conditions: 1. PRT + CCT 2. PRT + sham-CCT 3. CCT + sham-PRT 4. Sham PRT + sham-CCT	RCT double-blind	100 MCI	RS fMRI, seed (bilateral hippocampus and PCC) connectivity Pre-/Post-training	Bilateral hippocampi/PCC functional connectivity changes; cortical atrophy changes; performance improvement at the cognitive assessment (ADAS-Cog, Memory Awareness Rating Scale and Memory Complaint Score)	Group X Time/PRT vs non-PRT: Improvement on ADAS-Cog Group X Time/CCT vs on-CCT: no decline on memory domain	Decreased	Group X time/PRT: connectivity between PCC, L inferior temporal lobe and anterior cingulate cortex; and between hippocampus and R inferior temporal lobe. Group X time/CCT: connectivity between PCC, L superior frontal lobe and anterior cingulate cortex. Group X time/combined vs single intervention: connectivity between PCC and anterior cingulate cortex	In CCT, increased connectivity between hippocampus and L superior frontal; higher memory performance
							Increased	Group X time/PRT: connectivity between hippocampus and R middle frontal. Group X time/CCT: connectivity between hippocampus and L superior frontal lobe. Group X time/combined vs single intervention: connectivity between hippocampus, anterior cingulate cortex, and R superior frontal lobe	
Train the Brain Consortium 2016 [69]	7-month multidomain training, including cognitive, physical exercise and music therapy. Control group: MCI receiving usual care	RCT single-blind	113 MCI (different subtypes)	Visuo-spatial attention task At baseline At 7 months At 19 months	Task-related whole-brain functional activity changes; hippocampal cortical atrophy changes; white matter hyperintensities changes; performance improvement at cognitive assessment (ADAS-Cog)	Improvement on ADAS-Cog, on the immediate recall of the Rey-Osterrieth Complex Figure and on phonemic fluency. No improvement at the task	Unchanged	No pre-/post-training effect	–
Van Paasschen et al., 2013 [58]	8-week cognitive rehabilitation training (1 h sessions, 3 strategies for acquiring new information: verbal and visual mnemonics, semantic elaboration, and expanding rehearsal) Control: relaxing therapy and no training	RCT open-label	19 mild AD	Unfamiliar face-name pairs association task Pre-/Post-training	Task-related whole-brain functional activity changes; performance improvement at fMRI task, occupational assessment and mood (COPM and HADS)	Improvement on behavior assessed with the COPM. No improvement at the task	Decreased	Pre-/post-training, AD: Encoding: R insula	–
							Increased	Pre-/post-training, AD: Recognition: bilateral insula and angular gyrus, L middle frontal gyrus	
Wells et al., 2013 [64]	Mindfulness-based stress reduction (30 min/day, once a week for 8 weeks, 2 h each session + home practice). Control group: MCI receiving usual care	RCT single-blind	14 MCI	RS fMRI Pre-/Post-training	DMN/hippocampal functional connectivity changes; hippocampal atrophy changes and changes on ADAS-Cog	No significant changes on ADAS-Cog	Increased	Group X time, training vs control: connectivity between PCC and bilateral medial prefrontal cortex and between PCC and L hippocampus	–

*AD* Alzheimer’s disease, *ADAS-cog* Alzheimer’s Disease Assessment Scale-cognitive subscale, *COPM* Canadian Occupational Performance Measure, *CCT* computerized cognitive training, *DAD* Disability Assessment for Dementia, *DMN* default mode network, *FLSA* functional living skills, *fMRI* functional magnetic resonance imaging, *HADS* Hospital Anxiety and Depression Scale, *HC* healthy controls, *L* left, *MCI* mild cognitive impairment, *MST* multidimensional stimulation group therapy, *NPI* Neuropsychiatric Inventory Scale, NRCT nonrandomized controlled trial, *PCC* posterior cingulate cortex, *PRT* progressive resistance training, *R* right, *RAVLT* Rey auditory verbal learning test, *RBANS* Repeatable Battery for the Assessment of Neuropsychological Status, *RCPM* Raven’s Colored Progressive Matrices, *RCT* randomized controlled trial, *RS fMRI* resting state fMRI, *SF-36* Short Form 36 healthy survey questionnaire

#### Summary

Studies on cognitive rehabilitation proposed different types of training such as verbal and visual encoding, retrieval and mnemonic association strategies, auditory-verbal discrimination, mindfulness, singing therapy, reality orientation exercises, and occupational/recreational therapy. Studies investigating the effects of physical therapy were based on aerobic and progressive resistance training. While physical training lasted usually about 3 months, the cognitive and combined approaches presented greater duration variability (from 2 weeks to 7 months). Overall, both MCI and AD patients took advantage from cognitive training while only MCI patients seemed to benefit from physical therapy. The adopted fMRI approaches were resting-state fMRI (23%), or task-based fMRI (77%) using memory paradigms such as encoding, retrieval, association, and discrimination tasks (54%), visuo-spatial attention (8%), and verbal paradigms (15%). Due to the intensity of the programs and/or the difficulty of the proposed fMRI tasks, most of these studies focused on MCI rather than AD patients. A summary of findings is difficult due to the heterogeneity of training and task selection. However, it emerges that cognitive, physical, or combined training are mainly associated with enhanced brain activity or connectivity in trained patients with concomitant improvement in specific cognitive functions.

The effects of cognitive rehabilitation have been assessed with fMRI tasks in the majority of studies. After 2 months of training on strategies for acquiring new information, mild AD patients showed an increased activity in the frontoparietal areas and insula during an unfamiliar face-name association task [58]. Using singing training for 6 months, an improvement on daily living activities, behavior, and reasoning in mild-moderate AD patients, together with fMRI increased activation of the angular and lingual gyri during a Karaoke task, were observed [59]. In MCI patients, after an intense program of encoding/retrieval memory training, increased recruitment of frontotemporal areas, basal ganglia, and cerebellum was observed during a memory-encoding task [60], and of frontal, parietal, temporal, and occipital areas [61] and left hippocampus [62] during memory-association tasks. During the memory retrieval phase, in trained MCI patients, a specific relationship between the increased activity of the right inferior parietal lobule and the improved performance on verbal delayed recall was found [60]. After a 2-month computer-based program on auditory verbal discrimination, MCI patients showed increased activity in the left hippocampus during an auditory verbal task with a parallel improvement in memory performance as tested outside the scanner [63]. MCI patients showed an increased resting-state functional connectivity between the PCC and bilateral medial prefrontal cortex and between the PCC and left hippocampus after eight sessions of mindfulness-based stress reduction [64].

The effects of aerobic training have been assessed in MCI patients with both task-based and resting-state fMRI. After 3 months of moderate aerobic exercises, no specific effects on brain activations were observed using a semantic memory task [65], while an increased resting-state functional connectivity between the PCC and bilateral frontoparietal and temporal cortices, insula and cerebellum was observed in MCI cases [66]. An increased activity of the left caudate after regular high-intensity physical activity compared to low-intensity training was observed using a famous-name discrimination paradigm [67].

The efficacy of a combined (cognitive and physical) approach was investigated in three studies, which adopted multidimensional stimulation programs. In the first study, mild-moderate AD patients were involved in 30 training sessions [68]. After training, during a verbal fluency task, AD patients showed an increased recruitment of the bilateral superior temporal gyrus, right insula, and thalamus associated with improvement in global cognition [68]. In a second study, after a 7-month training, 113 MCI patients showed no specific training-related brain changes during a visuospatial attention task [69]. Finally, one study investigated the effect of 26 weeks of progressive resistance training and computerized cognitive training (CCT) in 100 MCI patients using resting-state fMRI [70]. Both trainings, as well as the combination of the two, were associated with changes in functional connectivity between the hippocampus, PCC, and frontotemporal regions [70]. Of note, increased connectivity between the hippocampus and left superior frontal cortex after CCT was associated with improved memory performance [70].

No study directly compared clinical and fMRI effect sizes in order to define the most powerful marker to monitor treatment efficacy.

#### Critical review

According to the Cochrane Collaboration’s tool for assessing risk of bias, seven studies (54%) were randomized but only four described clearly the randomization procedure and none the allocation. Five studies (38%) stated the blindness of participants and personnel; for two studies (15%) this information was unclear, and the other 6 (47%) were unblinded. Five studies (38%) reported the blindness of outcome assessment; for three studies (24%) this information was unclear, and the other five (38%) were unblinded. Two studies (15%) presented more than 20% drop-outs at short- and/or long-term assessments. All studies but one appropriately reported the primary and the secondary outcome measures of the investigation. A report of the final judgments for each selected article is shown in Fig. 2.

Common shortcomings of the reviewed works were regarding study design, patient selection, sample size, choice of outcome measures, management of drop-out cases, and fMRI methods.

In the following discussion, we underline the strengths and limitations of the reviewed studies and provide suggestions to overcome these issues.

## Discussion

### Patient selection, randomization, and allocation

The definition of the clinical population is a very critical point. Targets of the proposed treatments should be cases of prodromal or probable AD with a clinical diagnosis supported by biomarkers [71]. Over the last decades, the development of subject-selection strategies that strongly maximize the power of treatments by detecting target populations has been an important focus of large international studies such as the Alzheimer's Disease Neuroimaging Initiative [71]. Abnormal tau and amyloid β42 cerebrospinal fluid levels, baseline MRI atrophy, and apolipoprotein E ε4 status have been used as successful stratification strategies [72] and should be applied to define an early clinical population, such as MCI, or at-risk asymptomatic subjects. However, only a few of the reviewed studies [24, 26, 27, 29, 30] used biomarkers in the inclusion process and, for some others, the clinical features of the MCI population (if it was amnesic for instance) were also unclear. While selecting the study sample, the lack of a neat clinical definition together with the absence of biomarkers leads to underpowered and diluted findings.

In most of the reviewed articles, the randomization procedure was not performed due to the observational nature of the study design and to the absence of a group of placebo or active healthy controls. Although these studies observed an effect of the proposed treatments on the outcome measures, the authors cannot argue for a specific efficacy of the treatment itself since it could be due to the mere nature of the clinical intervention. The absence of a control condition also leads to the unblinding of participants and personnel; this is an additional confounding factor that affects the soundness of methods. On the other hand, many works, which declare to have adopted a randomized study design, failed to clearly describe the procedure of the subject randomization and allocation or introduced some *a priori* bias (such as *a priori* stratification of the sample by gender [68] or the decision of a disproportionate ratio of the group distribution [47]) that may affect the neutral distribution of subjects in the experimental groups.

We have the following suggestions: 1) the population should be well-defined clinically and the AD diagnosis should be biomarker-supported; and 2) randomization and allocation must follow recognized guidelines and should be clearly reported in the study description.

### Type, intensity, and duration of treatment

The persistence of effects, along with generalization of gain in everyday life, is the critical point of pharmacological and nonpharmacological therapies. The need of a long-term treatment to maintain positive effects engenders the problem of the treatment costs. It is noteworthy that the selection of the type, intensity, and duration of treatment has the potential to modulate its efficacy. For instance, studies comparing the clinical and fMRI effects of pharmacological treatments directly targeting synapses *versus* other types of therapies (e.g., inhibitors of cholinesterase enzymes) are lacking. In the case of nonpharmacological interventions, the long-term potential of the combination of cognitive and motor rehabilitation has been amply postulated in neurodegenerative disorders [73]; however, only two reviewed studies [68, 70] adopted this combined approach demonstrating its effect on cognitive and behavioral improvement even after 22 weeks [68]. The success of this last-mentioned study is also attributable to the nature of the proposed training, which involved both patients and caregivers thus guarantying a continuous care at home [68]. Furthermore, the different efficacy based on intensity of training has been poorly considered. This is important since in other conditions, such as in Parkinson’s disease, training on alternate days has been demonstrated to be more efficient compared to an intense (everyday) approach [74].

We have the following suggestions: 1) the selection of the type, intensity and duration of treatment is relevant and can modulate the long-term effect of intervention; 2) studies comparing the clinical and fMRI effects of pharmacological treatments directly targeting synapses *versus* other types of therapies are needed; and 3) in nonpharmacological interventions, studies aimed at assessing the efficacy of the cognitive and motor training combination as well as at establishing the optimal intensity of treatment are warranted.

### The choice of outcome measures

The main difficulty for these studies is to transfer outcome measures from the laboratory to real life. fMRI can contribute to this effort by identifying, through the task or using a resting-state approach, the brain regions or brain networks that are sensitive to treatment and that can predict the everyday activities for which treatment is likely to be effective.

However, building the proper fMRI task is challenging. First, cognitive fMRI experiments used to test behavioral longitudinal changes can be biased by learning effects, especially when the interval between pre- and post-treatment evaluation is short. The use of parallel versions of the same task avoids the detection of an improvement due to learning. In the majority of pharmacological studies, mainly the observational ones, the selected task is *disease-driven*, i.e., it has the aim to test the drug efficacy on cognitive domains known to be affected in AD such as episodic or semantic memory (encoding, recall, recognition, pair-association), visuo-spatial abilities, and auditory working memory. In the same way, when a resting-state approach is preferred, functional connectivity within the DMN, as the most affected network in AD, is usually the primary MRI outcome. Although this approach is understandable and driven by what we know about the AD pathology, it runs the risk of losing some important information on the treatment efficacy. With such *disease-driven* methods, mechanisms of compensation and brain reorganization in unaffected brain areas could not be captured. For this purpose, Dhanjal and Wise investigated the effect of cholinesterase inhibitors on non-DMN networks, such as salience and executive-control networks, in a group of AD patients to determine whether improving memory function via modulation of frontoparietal connectivity was a possible compensative mechanism [30]. The same strategy can be adopted by task-related fMRI designs, by observing if the activity of nonmemory brain circuits, such as those subtending selective attention and/or distracter inhibition, could modulate the improvement of the encoding processing and the successful recall.

In nonpharmacological studies, the selected task is usually *training-driven*, i.e., it is built to verify improvement in activity in brain regions known to subtend the training-related functions. For instance, in the Explicit-Memory Training proposed by Hampstead and colleagues [61], patients acquired mnemonic strategies using face-name associations and the fMRI task used the same paradigm to test its efficacy. However, there are some studies using generic fMRI tasks (such as verbal fluency) as well as clinical outcome measures assessing global cognitive status which are not specific and/or unrelated to the performed training. The risk in these latter cases is to observe changes in fMRI activity unrelated to the training.

Finally, no study to date has directly compared clinical/cognitive *versus* fMRI outcome effect sizes (only the relationship between these variables has been assessed) in order to define which marker is the most powerful in reflecting treatment effects over time.

We have the following suggestions: 1) parallel versions of the same fMRI task are needed to avoid learning effects; 2) a whole brain fMRI investigation is necessary to have a complete understanding on the effect of treatment in the whole brain; 3) training-driven tasks rather than global and unspecific tests are suggested as outcome measures in nonpharmacological studies; and 4) clinical/cognitive *versus* fMRI effect size comparisons should be provided.

### Incomplete outcomes, drop-out cases, and sample size

Incomplete outcome measures are often an important problem in these studies. The reasons for incomplete data or drop-outs are often related to the treatment itself (side-effects), but they could also be associated to the MRI environment (claustrophobia or difficulties lying down in the scanner during the entire duration of the protocol), technical MRI issues (motion artifacts or unrecorded behavioral performances during the task), patient difficulties in understanding and/or maintaining the task instructions, progression of the disease, changes in motivation, and lack of compliance. In aging and cognitive-impaired populations, cases of drop-out are frequent and should be considered during the recruitment phase by involving larger initial samples. In fact, if not considered, the consequences on the research protocol can be severe resulting in a reduction in the study power. For instance, Bokde and colleagues [47] enrolled 12 MCI patients in their trial and randomly assigned them to treated and placebo groups with a 2:3 ratio, respectively. Due to several drop-out cases, the placebo group finally included only two subjects and the analysis within this group was not statistically feasible [47]. Furthermore, negative findings are questionable in cases of a small sample size; for example, McGeown and colleagues [36] who reported no efficacy of 20 weeks of treatment with donepezil in a group of 12 AD patients on task-related fMRI activity and on behavioral performances.

By using a semi-cylindrical panel covering the patient’s body from the head to the knees (simulating the limited space in the scanner) together with a loud white noise through headphones (mimicking the noise of the scanner), Lorenzi and colleagues [56] performed a 9-min fMRI scan simulation during patient screening. This simple system tested the patient’s ability to rest, without moving, in an ‘unusual’ environment for the entire scan acquisition, thereby ensuring patient comfort and data quality. This simulation was useful for testing the patient tolerability to the MRI noise and environment, and for detecting the presence of claustrophobia and other behavioral complaints, such as agitation and anxiety, not identified during the interview with the caregiver but triggered during this ‘unusual’ situation. After the MRI simulation, 12 out of 28 moderate-to-severe AD patients did not pass the screening while the remaining all but one were successfully acquired and completed the study [56]. In addition, for some patients, task instructions could be difficult to understand and/or maintained during the sequence. Cognitive difficulties are likely to affect patient behavioral performances during the acquisition, and the fMRI signal could reflect a pattern unrelated to the investigated domain. A bias mitigation action could be to train the patient for several sessions prior to the MRI scan in order to assess task instruction comprehension and maintaining.

Finally, patient and caregiver motivation are also crucial for the success of the clinical trials. In the study of Baglio and colleagues [68], patients and caregivers underwent a multidimensional stimulation group therapy, which included 30 training sessions for the patient and an educational program to the caregiver to favor a long-term positive interaction with patients at home. The involvement of the caregivers was highly motivating with more than 80% of the initially recruited population still being part of the study at the 32-week clinical follow-up. However, in the same study, the fMRI part was apparently less ‘appealing’ since only 55% of the initial sample concluded the follow-up at week 10.

We have the following suggestions: 1) the statistical power of the study must be estimated, and larger samples should be recruited accounting for the attrition rate—multicenter collaborations could be an option to mitigate this issue; 2) results should be validated and tested using independent data; 3) simulations of MRI examination should be included in the patient screening phase for detecting cases of claustrophobia, behavioral complaints, or difficulties in lying down in the scanner; and 4) caregivers should get involved as much as possible in the study to increase patient compliance.

### Some MRI technical issues

Longitudinal MRI studies require monitoring of MRI data stability over time. The same MRI scanner should be used for all subjects for the entire duration of the study. The reproducibility of fMRI signal changes in young and old healthy individuals and in cognitively impaired subjects during memory tasks and resting state fMRI is only modest [75–78]. Thus, the MRI signal should be verified using pre- and postreproducibility studies. In this review, we noticed that only a few studies proposed two pretraining MRI scan sessions [29, 30, 60]. This is a key method for distinguishing brain changes related to repetition (a mere test-retest effect) from those associated with treatment or training. Unfortunately, the same studies [29, 30, 60] did not include control conditions, thus the test-retest study did not help to understand whether brain changes were specific to the treatment or training. Although a direct comparison between task-based and resting-state fMRI reproducibility has not been tested in any of the reviewed studies, the literature suggests that resting-state fMRI is more advantageous to provide reproducible patterns of fMRI connectivity over time and across scanner platforms since no special equipment is required and individuals do not have to be able to perform a cognitive task [79].

AD and MCI patients are known to have brain atrophy. However, only a few studies investigated cortical atrophy [27, 33, 45, 52], and only one study accounted for gray matter volume into the second-level fMRI analysis [55]. Partial volume effects can lead to a wrong interpretation of greater fMRI intensity in voxels with smaller proportions of gray matter with the risk of affecting group comparisons [80].

We have the following suggestions: 1) the same MRI scanner should be used for the entire duration of the study, and the stability of the MRI signal should be verified using pre/postreproducibility studies; and 2) second level analyses should take into account gray matter density at the voxel level.

## Conclusions

This critical review pointed at both strengths and caveats of the existing literature on the effects of pharmacological and nonpharmacological treatments on brain fMRI in AD and MCI. In general, although both task-based and resting-state fMRI have been valuable in detecting even subtle changes over a short period of treatment, current knowledge does not allow us to support fMRI as a suitable candidate outcome measure. Although a large amount of work has been done so far, there is an urgent need to increase the number and ameliorate the reliability of the studies by improving the soundness of the methods. We underline the importance of sample size and patient selection for increasing the statistical power, the need for validation and testing (using independent data), the appropriateness of the study design, and the ecological value of the interventions to increase the likelihood of transferability into daily life, and whole brain investigation in order to capture both pathological and compensatory mechanisms. Finally, existing literature suggests we care about the motivation of patients and caregivers in order to avoid drop-outs during the follow-up. Future larger studies with improved design will allow us to perform a meta-analysis, which is the best approach for providing conclusive information on fMRI as a relevant outcome measure.

## Abbreviations

AchEI: Acetyl-cholinesterase inhibitors
AD: Alzheimer’s disease
CCT: Computerized cognitive training
DMN: Default mode network
fMRI: Functional magnetic resonance imaging
MCI: Mild cognitive impairment
MRI: Magnetic resonance imaging
PCC: Posterior cingulate cortex
